# Whole‐genome sequencing identifies new candidate genes for nonobstructive azoospermia

**DOI:** 10.1111/andr.13269

**Published:** 2022-09-07

**Authors:** Agnieszka Malcher, Tomasz Stokowy, Andrea Berman, Marta Olszewska, Piotr Jedrzejczak, Dawid Sielski, Adam Nowakowski, Natalia Rozwadowska, Alexander N. Yatsenko, Maciej K. Kurpisz

**Affiliations:** ^1^ Institute of Human Genetics Polish Academy of Sciences Poznan Poland; ^2^ Scientific Computing Group IT Division University of Bergen Norway; ^3^ Department of Biological Sciences University of Pittsburgh Pittsburgh Pennsylvania USA; ^4^ Division of Infertility and Reproductive Endocrinology Department of Gynecology Obstetrics and Gynecological Oncology Poznan University of Medical Sciences Poznan Poland; ^5^ MNM Diagnostics Poznan Poland; ^6^ Department of Urology and Urologic Oncology in St. Families Hospital Poznan Poland; ^7^ Department of OB/GYN and Reproductive Sciences School of Medicine University of Pittsburgh Pittsburgh Pennsylvania USA

**Keywords:** biomarkers, infertility, nonobstructive azoospermia, spermatogenesis, whole‐genome sequencing

## Abstract

**Background:**

Genetic causes that lead to spermatogenetic failure in patients with nonobstructive azoospermia (NOA) have not been yet completely established.

**Objective:**

To identify low‐frequency NOA‐associated single nucleotide variants (SNVs) using whole‐genome sequencing (WGS).

**Materials and methods:**

Men with various types of NOA (*n* = 39), including samples that had been previously tested with whole‐exome sequencing (WES; *n* = 6) and did not result in diagnostic conclusions. Variants were annotated using the Ensembl Variant Effect Predictor, utilizing frequencies from GnomAD and other databases to provide clinically relevant information (ClinVar), conservation scores (phyloP), and effect predictions (i.e., MutationTaster). Structural protein modeling was also performed.

**Results:**

Using WGS, we revealed potential NOA‐associated SNVs, such as: *TKTL1, IGSF1, ZFPM2, VCX3A* (novel disease causing variants), *ESX1, TEX13A, TEX14, DNAH1, FANCM, QRICH2, FSIP2, USP9Y, PMFBP1, MEI1, PIWIL1, WDR66, ZFX, KCND1, KIAA1210, DHRSX, ZMYM3, FAM47C, FANCB, FAM50B* (genes previously known to be associated with infertility) and *ALG13, BEND2, BRWD3, DDX53, TAF4, FAM47B, FAM9B, FAM9C, MAGEB6, MAP3K15, RBMXL3, SSX3* and *FMR1NB* genes, which may be involved in spermatogenesis.

**Discussion and conclusion:**

In this study, we identified novel potential candidate NOA‐associated genes in 29 individuals out of 39 azoospermic males. Note that in 5 out of 6 patients subjected previously to WES analysis, which did not disclose potentially causative variants, the WGS analysis was successful with NOA‐associated gene findings.

## 1 INTRODUCTION

Male factor accounts for approximately 30%–50% of infertility cases, out of which up to 20% are considered to be idiopathic.^1^ Among those, the most severe form of male infertility characterized by the absence of spermatozoa in the ejaculate is called nonobstructive azoospermia (NOA).^2^

Causes of azoospermia include abnormal hormone levels, immune, or genetic factors. It can also be secondary to other systemic somatic abnormalities, such as testicular torsion or cryptorchidism. Consensus estimates that the interaction of over 2,000 genes involved during mitotic, meiotic, and postmeiotic stages leads to the generation of haploid gametes.^3^ Dysfunction of one of these stages can lead to spermatogenic arrest and azoospermia. However, precise addressing the genetic causes that lead to spermatogenesis failure is still required.

The current clinical diagnostic procedures for NOA include: semen evaluation, hormone analysis, and cytogenetic testing.^4^ Commonly, the analyses can be extended to genetic examination of Y microdeletions and obstructive azoospermia mutations in *CFTR* gene.^5^ However, there is no application of a clearly established clinical testing for NOA genes panel that should be routinely used for the diagnosis of NOA patients.

The increased availability of high‐throughput genomic sequencing analyses has helped to identify some causes of NOA development. Initially, we tested differentially expressed genes in patients with impaired spermatogenesis using testicular transcriptomic studies in spermatogenic failure. ^6^, ^7^, ^8^, ^9^ However, due to the high heterogeneity of NOA genetic background, the obtained expression results were not unequivocally causative genes or pathways specific for these patients. Therefore, we decided to apply high‐throughput sequencing to identify genetic abnormalities in NOA cases.

Recently, various genomic approaches were applied for NOA, as CGH, whole‐exome sequencing (WES).^10^, ^11^, ^12^, ^13^, ^14^, ^15^ Those studies identified novel genes responsible for the NOA phenotype, such as *GCNA* (germ‐cell nuclear antigen),^10^ *M1AP* (Meiosis 1 associated protein),^11^ *TDRD9* (Tudor domain containing 9),^16^ *GTF2H3* (general transcription factor TFIIH subunit 3),^14^ *MEI1* (meiotic double‐stranded break formation protein 1),^15^ *MEIOB* (meiosis specific with OB‐fold),^15^ and *TEX11* (testis expressed 11).^15^, ^17^ Unfortunately, the majority of NOA cases still remain undiagnosed, and their genomic etiology has been scarcely characterized.

WES provides a genetic diagnosis in only a limited number of cases.^18^ However, the application of whole genome sequencing (WGS) can provide a more powerful and accurate diagnosis due to the extended and balanced coverage, and the better resolution and detection of copy number gene variants.^19^, ^20^ This is important since a significant proportion of patients could have disease‐causing variants outside of the protein‐coding region, likely within regulatory regions of the genome, which can be assessed only using WGS, while the WES method typically omits genomic noncoding regions. These regions include noncoding regulatory elements (promotors, enhancers, insulators, silencers)^21^ and noncoding RNAs^22^ or deep intronic splicing variants.^23^ It was also reported that WGS is the best method for SNV detection in regard to sensitivity and coverage distribution.^24^ Single nucleotide variants (SNVs) have been reported to be associated with NOA, such as *MTRR*: c.537T>C (rs161870), c.1049A>G (rs162036), *PIWIL1*: c.1580G>A (rs1106042), *TAF4B*: c.1815T>C (rs1677016) and *SOX10*: c.927T>C (rs139884),^25^ *SPINK2*: c.56‐3C>G,^26^ *KDM3A* (rs34605051 and rs10246939), and *TEX15* (rs323344 and rs323345).^27^

Here, we present WGS to detect potential causative SNVs in patients with NOA (*n* = 39). Our study included 33 samples for which WGS was performed as the first line of molecular diagnosis, and 6 samples analyzed by WGS supporting the previously performed WES that did not disclose causative variants.

## 2 MATERIALS AND METHODS

### 2.1 Azoospermic patients, blood collection, and testicular biopsy

We collected whole blood and/or testicular samples from patients with NOA (*n* = 39), including the samples in which no mutations were previously identified by WES (*n* = 6).

All the patients were tested and did not show the following causes of infertility: abnormal karyotype, Y chromosome microdeletions, *CFTR* gene mutations, presence of antisperm antibodies, active or past orchitis, testicular torsion, cryptorchidism or varicocele.

Whole blood samples were collected into sterile single test tubes with EDTA (for DNA extraction) in a 5–10 ml volume. Testicular biopsy specimens (3–5 mm^3^) were collected in RNAlater solution (Ambion Life Technologies, Carlsbad, CA, USA) during a standard infertility workup that included a histopathological evaluation. Testicular samples were classified into four categories by histopathological image description: postmeiotic arrest (Johnsen score 6–9: 16 samples), meiotic arrest (Johnsen score 4–5: 3 samples), premeiotic arrest (Johnsen score 3: 3 samples), and Sertoli‐cell‐only syndrome (SCOS; Johnsen score 2: 10 samples), and are presented in Table S1.

This study was approved by the Local Bioethics Committee of Poznan University of Medical Sciences (Permission No. 1003/18), and all participants provided informed consent.

### 2.2 Controls–allelic frequencies out of over 140,000 individuals

Variant frequencies for WGS from healthy controls have been obtained from the GnomAD database (frequencies from 125,748 exomes and 15,708 genomes, http://gnomad.broadinstitute.org). In particular, we verified the respective frequency in the European population.

### 2.3 DNA extraction from blood and/or testicular samples

DNA isolation was performed according to the manufacturer's instructions. In the case of whole blood samples, the DNA IQ kit for Maxwell (Promega, Madison, WI, USA) was used, while for testicular samples, DNA was extracted using the AllPrep DNA/RNA/Protein Mini Kit (Qiagen, Hilden, Germany). DNA purity and concentration were measured using a spectrophotometer (NanoDrop 1000, Thermo Scientific, Waltham, MA, USA) and Quantus (Promega, Madison, WI, USA). Purified DNA samples were stored at −20°C until use.

### 2.4 Whole‐genome sequencing

WGS was performed using Illumina HiSeq X with the aim of obtaining coverage of at least x30 (100–120 Gb per sample). The quality of raw data was evaluated using the FastQC and MultiQC packages. Reads were aligned to the reference human genome GRCh37 using bwa‐mem aligner included in the Speedseq package.^28^ SNVs and small indels were called using freeBayes v0.9.21 (Speedseq‐var) as previously described.^29^ Variant files obtained from whole genome data were annotated using Ensembl Variant Effect Predictor.^30^ Results from all patients obtained in the form of tab separated text files were imported into R programming environment for filtering. To prepare Tables 1, 2, 3 the following filtering criteria were applied (Figure S1):

1. Variant type: nonsynonymous, nonsense or frameshift
2. Variant frequency in gnomAD VAF < 0.01 (variants with frequency gnomAD VAF < 0.001 were marked in tables with bold font)^31^
3. Recessive disease model (hemizygous, homozygote, or compound heterozygote)
4. Mutation Taster prediction “Disease causing”^32^
5. Expression in testis: NCBI gene and/or EMBL‐EBI (Ilumina Body Map)
6. Localization/expression of genes: human protein atlas (single cell type)
7. Potential function and relation with fertility/spermatogenesis: PubMed = Gene ID + “spermatogenesis” or “infertility” or “azoospermia” or “reproductive biology”
8. Clinically relevant information: dbSNP (https://www.ncbi.nlm.nih.gov/snp/), OMIM (https://www.ncbi.nlm.nih.gov/omim/), clinical variant status (ClinVar)

Following criteria 1 and 2, we obtained table with an average of 280 rare variants per individual which could have potential impact on protein function. Application of criteria 3–8 resulted in a short list of variants, which matches combined Tables 1, 2, 3. To illustrate variants in the context of our work, we applied literature review described below to divide obtained variants into medical context.

### 2.5 Structural protein modeling

Structural modeling was performed using Phyre2.^33^ Figures of the models of TKTL1, IGSF1, ZFPM2, and VCX3A were generated with the PyMOL Molecular Graphics System (Schrödinger). No postmodeling refinement was performed.

## 3 RESULTS

In this study, we applied WGS for 39 patients with nonbstructive azoospermia to identify novel NOA‐associated SNVs. For each patient, we determined approximately 280 rare variants (VAF < 0.01). We have found 8 potentially disease causing variants in 4 genes, followed by 30 variants in 20 genes that were previously linked to infertility, and 20 variants in 13 genes that have never been investigated with respect to male infertility but could be important in patients with NOA. We focused primarily on ultrarare variants with GnomAD MAF frequency < 0.001, which we highlighted in the tables (Table 1, 2, 3, Table S2). These variants were also filtered to search for direct or indirect relationships to spermatogenesis using databases such as PubMed (https://pubmed.ncbi.nlm.nih.gov/), UniProt (https://www.UniProt.org/), Ensembl (https://www.ensembl.org/index.html), the Human Protein Atlas (https://www.proteinatlas.org/), Gene Ontology (http://geneontology.org/) and MGI, Mouse Genome Informatics (http://www.informatics.jax.org/) (Tables 1, 2, 3, Table S4). We have also screened literature concerning panel of known NOA‐linked genes,^34^, ^35^ including also information about known chromosomal translocations or mutations (Tables 1, 2, 3, Tables S2 and S3). We focused on very rare variants in genes that have never been studied in NOA but are mostly expressed in the testis and/or the endocrine system. We screened all the chromosomes to look for the hemizygous, homozygous or compound heterozygous SNVs in genes that might be potentially NOA‐associated (including also known NOA‐genes), where the most notable were above all X‐linked variants (Tables 1, 2, 3, Table S2). We have also collated variants in known infertility‐related genes, but because the impact of the variants were “LOW”/“MODERATE” or were single heterozygous only, therefore these genes are not principal goal of our investigation and were presented in Table S3.

### 3.1 Novel causative variants identified in patients with nonobstructive azoospermia

**FIGURE 1:**
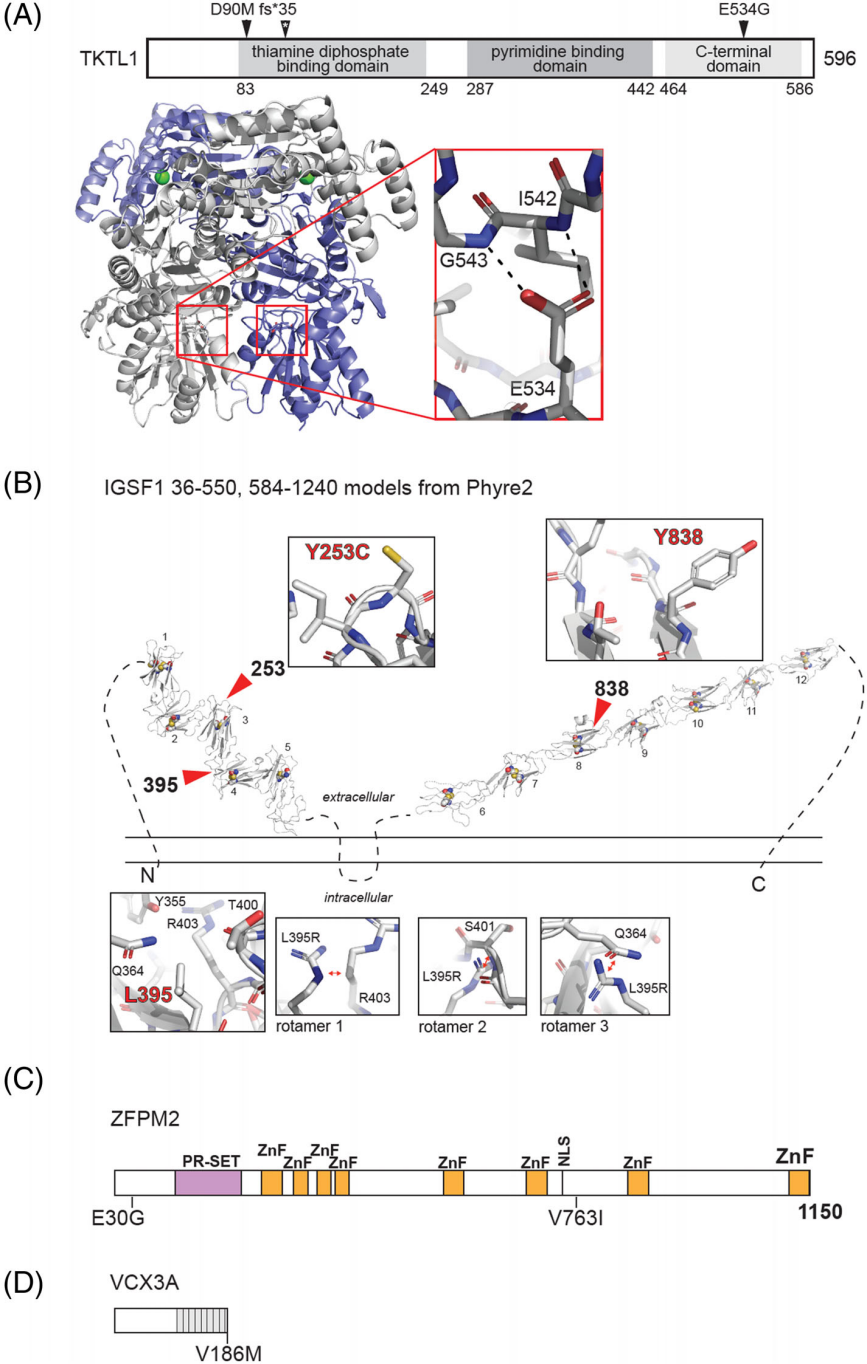
Structural modeling of TKTL1, IGSF1, ZFPM2, and VCX3A reveals potential mechanisms related to disease. (A) Diagram of TKTL1 with the indicated domains.^47^, ^48^ Numbers below represent amino acid positions in the primary structure. Above, positions of mutations of interest are shown. *Location of premature truncation due to the frameshift associated with D90M. Below this scheme, the model of the homodimer of TKTL1 in the schematic representation is shown. One monomer is colored blue, and the other is colored white. Green spheres represent positions of chelation of the calcium ion cofactor. Red boxes show the position of E534 in the context of the putative homodimer. The inset shows the position of E534 and the putative hydrogen bonds of its side chain carboxyl group with the backbone of amides of residues 542 and 543. (B) Phyre2‐^33^‐based structural homology modeling of IGSF1. The predicted orientation of IGSF1 relative to the plasma membrane is indicated with modeled tandem immunoglobulin‐like C2‐set motifs shown in cartoon view and numbered.^47^, ^48^ Locations of residues of interest are indicated. Disulfide bonds are shown as sticks, and unmodeled loops are shown as dotted black lines. Insets show the environment around labeled residues. The most energetically favorable rotamers of L395R are also shown. Red arrows indicate putative clashes between the mutant arginine residue and surrounding amino acids. (C) Linear organization of ZFPM2. ZnF, zinc finger; PR‐SET, PR‐SET domain. Below, residues of interest are indicated along the linear diagram. (D) Linear organization of VCX3A. The eight L–S–Q–E–S–[E or Q]–V–E–E–P sequence motifs are shown as light gray boxes

We identified two ultrarare variants (c.268_268delG and c.1601A>G) in the *TKTL1* gene (*locus* Xq28), and in silico analysis showed a disease‐causing effect in MutationT@ster (Table 1). This gene encodes a transketolase‐like protein 1, TKTL1, one out of the three transketolase proteins encoded in the human genome. High expression of this gene is exclusively present in the testis, with a 169.7% transcripts per million (TPM) value (https://www.gtexportal.org/home/gene/ENSG00000007350.16). The three‐dimensional homology model TKTL1 was modeled with SWISS‐MODEL^36^, ^37^ and Phyre2.^33^ TKTL1, likely a homodimer, is predicted by sequence conservation to have a thiamine phosphate binding domain, a pyrimidine binding domain, and a C‐terminal domain. Both homology modeling servers returned equivalent structures using transketolases as template models, including two high‐resolution structures of human transketolase;^38^, ^39^ the model generated by Phyre2^33^ covered 97% of all residues in TKTL1 with 99% confidence. The models of TKTL1 were consistent with homodimerization and a published homology model (Figure 1A).^40^ These models could easily be used to predict the functional implications of mutations of interest. The c.268_268delG variant was detected in patient with MA arrest and led to the following change in the protein: p.Asp90Met fs*35 (Table 1, Figure 1A, Figure S2A,B). This variant not only results in the mutation of a negatively charged aspartic acid residue to a hydrophobic amino acid, but also truncates most of the protein (Table 1, Figure 1A). The second variant, c.1601A>G, led to the amino acid change p.Glu534Gly in the C‐terminal domain, suggesting that this mutation destabilizes the fold in this domain (Table 1, Figure 1A, Figure S2A,B). For both patients, we observed lack of expression of the *TKTL1* gene at both mRNA and protein levels (Figure S2C,D).

**TABLE 1:** Identified novel disease causing variants in genes without previous evidence in nonobstructive azoospermia (NOA)

						**Controls from dbSNP database**		
**Patient no./Phenotype**	**Gene**	**Change in DNA (according to GRCh37)**	**Protein/RNA change**	**Mutation taster prediction**	**SIFT/PolyPhen/PhyloP100/CADD**	SNP	Alle frequency gnomAD	**Chromosomal translocations (H)/ other known mutations (H)/ mouse KO–MGI (M)**
53/P213 MA arrest	*TKTL1*	**chrX:153537712_** **153537712delG** ***c.268_268delG*** ***g.13689_13689delG***	p.Asp90Met fs*35	disease causing	–/–/–/–	–	**Variant not found**	MGI ID:6194752 Reproductive system: normal (J:234235)
34P Premeiotic arrest	*TKTL1*	**chrX:153556287A>G** ***c.1601A>G*** ***g.32264A>G***	p.Glu534Gly	disease causing	Deleterious (0.01)/probably_damaging(0.998)/8.426/ 27.3	–	**Variant not found**	
31L SCOS	*IGSF1*	**chrX:130417148T>C** ***c.758A>G*** ***g.116530A>G***	p.Tyr253Cys	Disease causing	Tolerated (0.17)/probably_damaging(0.998)/0.807/23.3	rs201801732	**European (non‐Finnish): 0.0005176** **Total 0.0002823**	OTHER KNOWN MUTATIONS (H): c.2284_2285insA MALE INFERTILITY MGI ID:2671059 Reproductive system: normal (J:190867)
41P MA arrest	*IGSF1*	**chrX:130419111G>C** ***g.114567C>G***	Intron	Disease causing	Deleterious (0)/probably_damaging(0.996)/1.742/18.54	rs142822502	**European (non‐Finnish): 0.001115** **Total 0.0006002**	
45L Postmeoitic arrest	*IGSF1*	**chrX:130410982A>C** ***c.2554T>G*** ***g.122696T>G***	p.Tyr838Asp	Disease causing	Deleterious(0)/probably_damaging(0.998)/4.736/26.1	–	**Variant not found**	
10L Postmeoitic arrest	*ZFPM2/* *FOX2*	chr8:106431420A>G HETERO *c.89A>G* *g.100501A>G*	p.Glu30Gly	Disease causing	Deleterious(0.03)/probably_damaging(0.998)/7.011/ 25.1	rs121908601	European (non‐Finnish): 0.004557 Total: 0.002691	OTHER KNOWN MUTATIONS (H): p.S402R and p.R260Q p.M544I DSD MGI ID: 5749460 Reproductive system: Heterozygotes are normal (J:216295) * No information about homozygotes and compound heterozygous
		chr8:106814597G>A HETERO *c.2287G>A* *g.483678G>A*	p.Val763Ile	Disease causing	Tolerated(0.05)/benign (0.084)/4.06/ 15.20	rs117908591	European (non‐Finnish): 0.004157 Total: 0.002646	
9L Postmeoitic arrest	*ZFPM2* *FOX2*	chr8:106814597G>A HOMO *c.2287G>A* *g.483678G>A*	p.Val763Ile	Disease causing	Tolerated(0.05)/benign (0.084)/4.06/15.20	rs117908591	European (non‐Finnish): 0.004157 (only 2 HOMO) Total: 0.002646 (only 3 HOMO)	
60P SCOS	*VCX3A*	**chrX:6451791C>T** ***c.556G>A*** ***g.1369G>A***	p.Val186Met	Disease causing	Deleterious(0)/probably_damaging(0.729)/0.608/18.62	rs74393938	**European (non‐Finnish): 0.0005998** **Total: 0.0007126**	TRANSLOCATION/OTHER KNOWN MUTATIONS (H): A novel 4.8 Mb deletion involving *VCX3A* gene together with others KALLMANN SYNDROME MGI (M): N/A

*Note*: Ultra‐rare variants (<0.001) are shown in bold. H, human; M, mouse; MGI, mouse genome informatics.

We also identified four likely pathogenic variants in the *IGSF1* gene (c.758A>G, c.1184T>G, g.114567C>G, c.2554T>G) (*locus* Xq26.1) in 4 out of 39 patients. In 3 variants, the predicted in silico analysis showed that their protein features were affected and that splice site changes with disease‐causing effects in MutationT@ster (Table 1). This gene encodes a plasma membrane Ig superfamily glycoprotein that consists of 12 C2‐type Ig loops, a transmembrane domain and a cytoplasmic domain (Figure 1B).^41^ The product of this gene is probably involved in hormonal regulation and circulating thyroid stimulating hormone (TSH).^42^ High expression of this gene is especially present in the pituitary gland but is also observed in the testis, with a 9.798 TPM value (https://www.gtexportal.org/home/gene/ENSG00000147255). We performed homology modeling using the Phyre2 server in both normal and intensive modes.^33^ Amino acids 36‐550 of IGSF1 isoform 1 were modeled with 99% confidence to contain five immunoglobulin (Ig)‐like constant2‐set (C2‐set) domains in tandem (Figure 1B, Figure S3A); residues 571 to the C‐terminus were modeled with 99% confidence to be comprised of seven Ig‐like C2‐set motifs (Figure 1B, Figure S3A). Very little of the sequence comprised disordered residues (Figure S3B). Tandem Ig‐like folds are predicted to be extracellular and separated by a two‐transmembrane helix domain.^33^ The Ig‐like fold is a common receptor fold comprised of seven antiparallel β‐strands arranged using Greek key topology.^43^ Characteristically for Ig folds, each domain was modeled with one disulfide bond;^43^ these residues were not included as restraints in the undertaken modeling, thereby increasing the reliability of the model. While the individual domains have been modeled with high confidence, one must be careful not to overinterpret the interdomain interactions. The first two *IGSF1* variants are located in the start region of the sequence. Variant c.758A>G was identified in patient P31L with SCOS and led to an amino acid change (p.Tyr253Cys) in the third C2‐type Ig loop, whereas variant c.1184T>G was observed in a patient with postmeiotic arrest (P10L) and introduced an amino acid change (p.Leu395Arg) in the fourth C2‐type Ig loop (Figure 1B). The next identified variant, c.2554T>G, observed in another patient with postmeiotic arrest (P45L), was located in the eighth C2‐type Ig loop of the extracellular domain and led to an amino acid change (p.Tyr838Asp) (Figure 1B). In patient P41P with spermatogenesis failure at the meiotic stage, the variant g.114567C>G in the intronic region of the *IGSF1* sequence was found and was predicted to be disease causing (Table 1). Three out of four variants in the *IGSF1* sequence have not been previously reported in the ClinVar database. Only c.1184T>G variant was reported as benign but lacked a detailed phenotypic description.

Similarly, we observed potentially pathogenic variants associated with NOA in the *ZFPM2* gene (*locus* 8q23.1), reported in disorder of sex development (DSD).^44^ This gene encodes the zinc finger‐containing protein, a member of the FOG family regulators of transcription factors.^45^ The ZFPM2 protein appears to be able to directly interact with GATA (GATA binding protein 1); therefore, it may either enhance or repress the ability of GATA transcription factors to stimulate expression of its target genes, depending on their promoter section context.^44^ It is also required in the gonadal differentiation process and is proposed to regulate *SRY* expression.^46^ The *ZFPM2* gene is strongly expressed in the ovary with a TMP value of 26.96, but its expression also occurs in the testis (TMP = 3.299). Based on the sequence, ZFPM2 is predicted to have a PR‐SET domain and eight zinc finger motifs^47^, ^48^ separated by regions that can be described as unstructured, intrinsically disordered, or low‐complexity (Figure S3). Disorder prediction and ab initio modeling^49^, ^50^, ^51^ suggested that most of the *N*‐terminal region of the protein is disordered with the exception of a short ∼10‐residue helix around residue 60 (Figure 1C, Figure S3). In our experiments, we identified the following variants of the *ZFPM2* gene in azoospermic patient P10L with postmeiotic arrest: compound heterozygous variants c.89A>G and c.2287G>A, which introduced amino acid change p.Glu30Gly and p.Val763Ile, respectively (Table 1, Figure 1C). One of these variants (c.2287G>A; p.Val763Ile) was also observed in another patient with postmeiotic arrest (P9L) as a homozygous variant (Table 1). In silico analysis predicted all of these variants to be disease causing (Table 1). Variant c.89A>G was previously associated with 46,XY sex reversal 3 (OMIM: 612965).

Moreover, we identified the ultrarare variant c.556G>A within the *VCX3A* gene (*locus* Xp22.31) in a patient with SCOS (Table 1), which is expressed exclusively in the testis (https://gtexportal.org/home/gene/ENSG00000169059.12). This gene belongs to the VCX gene family and encodes small and highly charged proteins of unknown function.^52^ All secondary and tertiary modeling programs tested^33^, ^47^, ^49^, ^50^ revealed >90% confidence that the entire VCX3A protein was disordered, with the exception of residues 28–35 (Figure S3). This protein has eight tandem repeats of L–S–Q–E–S– [E or Q] –V–E–E–P between positions 104–183 (Figure 1D),^48^ and these repeats are predicted to be unstructured or disordered (Figure S3).

### 3.2 Rare variants identified in individual nonobstructive azoospermia samples in genes previously associated with infertility

This group included new variants in known genes related to somatic chromosomes associated with infertility such as *TEX14* (locus 17q22; c.2285_2288delAGAA: homozygous)*, DNAH1* (locus 3p21.1; c.8455G>A, c.11494_11495delinsAG: compound heterozygous)*, FANCM* (locus 14q21.2; g.19521G>A, c.2081T>G: compound heterozygous), *QRICH2* (locus 17q25.1; c.1888_1889delinsGC: homozygous)*, FSIP2* (locus 2q32.1; c.1970A>T, c.12230C>T: compound heterozygous)*, PMFBP1* (locus 16q22.2; c.1654C>G, c.646C>T: compound heterozygous)*, MEI1* (locus 22q13.2; c.1546A>G, c.3542G>A: compound heterozygous)*, PIWIL1* (locus 12q24.33; g.11346C>T, c.1255C>T: compound heterozygous)*, WDR66* (locus 12q24.31; c.562C>T, c.2578C>T: compound heterozygous) (Table 2, Tables S2–S4) and 14 rare variants in genes located on chromosomes X or Y, which have been previously observed in infertility (Table 2, Tables S2–S4). Among these variants we noted rare variants in *ESX1* gene (c.1040C>G, c.1042C>G). In this gene, another mutation (c.1094C >G, c.1096C>G) was reported in the Han Chinese population as a high risk factor for NOA, likely by affecting cell cycle control.^53^ *TEX13A* (c.596G>A) in which translocations including this gene were reported as genetic risk factors for azoospermia;^54^ *KCND1* (c.1396C>G; c.1723T>G)—this gene is involved in volume regulation by murine spermatozoa;^55^ *KIAA1210* (c.4207G>T; c.1214G>A)—it was suggested that KIAA1210 is important for regulating the dynamic change of chromatin structures during spermiogenesis;^56^ *DHRSX* (g.14068A>C)—it was reported that aberrations in the region including this gene may result in the deficiency of X–Y pairing or recombination, ultimately leading to the spermatogenic failure;^57^ *ZMYM3* (c.2367C>G)—knockout of *Zmym3* was observed in mice with arrested spermatogenesis at meiotic metaphase;^58^ *FAM47C* (c.1826A>G) —this gene was described as a possible cause of severe oligozoospermia;^59^ *FANCB* (c.1769T>C)—this gene is involved in the germ cell development at critical stages^60^; *USP9Y* (c.3293C>T) and *ZXF* (c.465T>A) —these genes were previously reported in NOA patients (details are presented in Table 2, Tables S2–S4). Another example of a gene that has been previously described in the male infertility context and aberrant DNA methylation is *FAM50B* (*locus* 6p.25.2),^61^ which was identified in a patient with postmeiotic arrest (P9L) following homozygous variant c.293G>A (Table 2, Table S2).

**TABLE 2:** Identified variants in previously determined genes associated with infertility and/or nonobstructive azoospermia (NOA)

						**Controls from dbSNP Database**		
**Patient no/ Phenotype**	**Gene**	**Change in DNA (according to GRCh37)**	**Protein/RNA change**	**Mutation Taster prediction**	**SIFT/PolyPhen/ PhyloP100/CADD**	SNP	Allele frequency GnomAD	**Chromosomal translocations (H)/ other known mutations (H)/mouse KO ‐ MGI (M)**
10L Postmeoitic arrest	*ESX1*	**chrX:103495090G>C** ***c.1040C>G*** ***g.4525C>G***	p.Pro347Arg	Polymorphism: amino acid sequence changed protein features (might be) affected splice site changes	Tolerated(0.81)/ –/0.956/16.03	rs200088361	**European (non‐Finnish): 0.0002010** **Total: 0.0008919**	OTHER KNOWN MUTATIONS (H): c.1094C >G and c.1096 C > G Azoospermia MGI ID (M):2153820 Reproductive system: N/A
		**chrX:103495088G>C** ***c.1042C>G*** ***g.4527C>G***	p.Leu348Val	Polymorphism: amino acid sequence changed protein features (might be) affected	Tolerated(1)/benign(0)/−2.38/0.004	rs76090537	**European (non‐Finnish): 0.0001976** **Total: 0.0009192**	
33P Postmeoitic arrest	*ESX1*	**chrX:103495090G>C** ***c.1040C>G*** ***g.4525C>G***	p.Pro347Arg	Polymorphism: amino acid sequence changed protein features (might be) affected splice site changes	Tolerated(0.81)/–/0.956/16.03	rs200088361	**European (non‐Finnish): 0.0002010** **Total: 0.0008919**	
		**chrX:103495088G>C** ***c.1042C>G*** ***g.4527C>G***	p.Leu348Val	Polymorphism: amino acid sequence changed protein features (might be) affected	Tolerated(1)/benign(0)/−2.38/0.004	rs76090537	**European (non‐Finnish): 0.0001976** **Total: 0.0009192**	
P235 Postmeiotic arrest	*TEX13A*	**chrX:104464282C>T** ***c.596G>A*** ***g.1077G>A***	p.Gly199Glu	Polymorphism: amino acid sequence changed protein features (might be) affected splice site changes	Deleterious(0)/benign(0)/ −0.312/23.4	–	**European (non‐Finnish): 0.000** **Total: 0.000**	TRANSLOCATION (H): one patient 46,Y,t(X;19)(q22;q13.3) and the other with 46,Y,t(X;8)(p22;q11) Azoospermia MGI ID:5708278 Reproductive system: normal (J:279039)
P254 N/A	*TEX14*	**chr17:56676418_56676421delTTCT** **HOMO** ***c.2285_2288delAGAA*** ***g.92996_92999delAGAA***	p.Gln762Argfs*31	Disease causing	–/–/–/–	–	**Variant not found**	OTHER KNOWN MUTATIONS (H): Compound heterozygote: NM_001201457.1:c.2303_2306del; p.(Gln768ArgfsTer31) NM_001201457: c.(554+1_555‐1)_(3378+1_3378‐1)del p. 185del941aaAZO Homozygous: NM_001201457.1:c.3454C>T; p. (Arg1152Ter) AZOOSPERMIA MGI ID: 1933227 Reproductive system: spermatogenic failure 23 (J:107660)
P‐76‐2014 N/A	*DNAH1*	**chr3:52418934G>A** **HETERO** ***c.8455G>A*** ***g.68600G>A***	p.Gly2819Arg	disease causing	tolerated(0.36)/benign(0.015)/ 5.04/23.7	rs372068387	**European (non‐Finnish): 0.00004694** **Total: 0.00005725**	OTHER KNOWN MUTATIONS (H): Heterozygous: c.5626G>C c.7066C>T c.11726_11727del c.8322+3del c.6446T>G c.11412del c.7201del c.7205C>A ASTHENOSPERMIA OR AZOOSPERMIA MGI ID:107721 Reproductive system: spermatogenic failure 18 (J:69800)
		**chr3:52430697_** **52430698delinsAG** **HETERO** ***c.11494_11495delinsAG*** ***g.80363_80364delinsAG***	p.His3832Ser	probably disease causing	deleterious(0)/ benign(0)/–/–	–	**European (non‐Finnish): 0.00008570** **Total: 0.00004635**	
43P SCOS	*FANCM*	**chr14:45624663G>A** **HETERO** ***g.19521G>A***	Intron	Disease causing	–/–/3.393/33	–	**Variant not found**	OTHER KNOWN MUTATIONS (H): compound heterozygous: c.1778delG p.R593Qfs*76 c.1663G>T p.V555F homozygous: c.1972C>T p.R658X AZOOSPERMIA MGI ID:2442306 Reproductive system: Azoospermia (J:301639)
		**chr14:45639870T>G** **HETERO** ***c.2081T>G*** ***g.34728T>G***	p.Leu694*	Disease causing	–/–/5.316/36	–	**Variant not found**	
38L MA arrest	*QRICH2*	**chr17:74288421_74288422delinsGC** **HOMO** ***c.1888_1889delinsGC*** ***g.15340_15341delinsGC***	p.Ile630Ala	Polymorphism: amino acid sequence changed protein features (might be) affected splice site changes	Tolerated(0.71)/benign(0)/ −6.409/ 0.168	–	**European (non‐Finnish): 0.00007920** **Total: 0.0005520**	OTHER KNOWN MUTATIONS (H): p.Cys1644AlafsTer52 MGI:2684912 Male infertility (J:270177)
50L SCOS								
63P/P219 Postmeiotic arrest								
P78/2014 N/A	*FSIP2*	chr2:186653566A>T HETERO *c.1970A>T* *g.50212A>T*	p.Tyr657Phe	Polymorphism: amino acid sequence changed	Deleterious(0.03)/possibly_damaging(0.882)/1.293/17.72	rs111265848	European (non‐Finnish): 0.001878 Total: 0.001228	OTHER KNOWN MUTATIONS (H): c.[910delC] c.[2282dupA] c.[1606_1607insTGT; 1607_1616delAAAGATTGCA] c.[16389_16392delAATA] Asthenozoospermia c.8030_8031insA, p.T2680Nfs*9 Asthenoteratospermia MGI ID:2664111 Reproductive System: Spermatogenic failure 34 (J:307606)
		chr2:186665996C>T HETERO *c.12230C>T* *g.62642C>T*	p.Ser4077Phe	Polymorphism: amino acid sequence changed protein features (might be) affected splice site changes	Tolerated(0.19)/probably_damaging(0.948)/0.562/23.4	rs113773415	European (non‐Finnish): 0.001858 Total: 0.001214	
65P SCOS	*USP9Y*	**chrY:14898465C>T** ***c.3293C>T*** ***g.14898465C>T***	p.Ala1098Val	MutationTaster can thus neither generate the correct protein sequence nor conduct the necessary tests to make a prediction. Other prediction algorithms shown following results: SIFT: tolerated(0.05) PolyPhen: benign(0.041)	Tolerated(0.05)/benign(0.041)/5.735/17.92	rs202095134	**European (non‐Finnish): 0.0003990** **Total**: **0.0002359**	OTHER KNOWN MUTATIONS (H): intron 7‐ GTAA (splice donor) deleted AZOOSPERMIA
41P MA arrest	*PMFBP1*	chr16:72164240G>C HETERO *c.1654C>G* *g.46538C>G*	p.Leu552Val	Polymorphism: amino acid sequence changed protein features (might be) affected splice site changes	Deleterious(0)/probably_damaging(0.994)/1.797/ 22.2	rs144092086	European (non‐Finnish): 0.004382 Total: 0.002835	OTHER KNOWN MUTATIONS (H): homozygous: c.301A>C (p.T101P) ACEPHALIC SPERMATOZOA SYNDROME MGI ID:1930136 Reproductive system: ACEPHALIC SPERMATOZOA (J:303070)
		chr16:72170469G>A HETERO *c.646C>T* *g.40309C>T*	p.Arg361Trp	Polymorphism: amino acid sequence changed	Deleterious(0)/probably_damaging(0.966)/1.101/ 23.	rs147286664	European (non‐Finnish): 0.004221 Total: 0.002758	
31L SCOS	*MEI1*	**chr22:42141896A>G** **HETERO** ***c.1546A>G*** ***g.46394A>G***	p.Ile516Val	Polymorphism: amino acid sequence changed splice site changes	Tolerated(1)/benign(0)/0.446/10.71	rs1230316113	**European (non‐Finnish): 0.000** **Total: 0.000004034**	OTHER KNOWN MUTATIONS (H): homozygous: c.C3307T (p.R1103W) AZOOSPERMIA WITH MEIOTIC ARREST MGI ID: 3028590 AZOOSPERMIA (J:103876)
		**chr22:42191422G>A** **HETERO** ***c.3542G>A*** ***g.95920G>A***	p.Arg1181Gln	Polymorphism: amino acid sequence changed splice site changes	Tolerated(0.57)/benign(0.007)/0.827/18.93	rs767330085	**European (non‐Finnish): 0.00002663** **Total: 0.00001206**	
P235 Postmeiotic arrest	*PIWIL1*	chr12:130833777C>T HETERO *g.11346C>T*	intron	Polymorphism: protein features (might be) affected splice site changes	–/–/−0.796/ 0.573	rs117552783	European (non‐Finnish): 0.01521 Total: 0.009547	OTHER KNOWN MUTATIONS (H): c.1580G>A (rs1106042) AZOOSPERMIA MGI ID:1928897 AZOOSPERMIA (J:182800)
		chr12:130839516C>T HETERO *c.1255C>T* *g.17085C>T*	p.Arg419Cys	disease causing	Deleterious(0.05)/benign(0.443)/0.775/23.9	rs35540071	European (non‐Finnish): 0.0008595 Total: 0.001733	
65P SCOS	*WDR66*	chr12:122361711C>T HETERO *c.562C>T* *g.5944C>T*	p.Arg188Trp	Polymorphism: protein features (might be) affected splice site changes	Deleterious(0.01)/benign(0)/0.848/12.11	rs34703321	European (non‐Finnish): 0.008113 Total: 0.004806	OTHER KNOWN MUTATIONS (H): homozygous 8.4 kb intragenic deletion encompassing *WDR66* ASTHENOZOOSPERMIA
		chr12:122404946C>T HETERO *c.2578C>T* *g.49179C>T*	p.Arg860Cys	disease causing	Deleterious(0.03)/possibly_damaging(0.453)/1.62/24.7	rs146415200	European (non‐Finnish): 0.008128 Total: 0.004901	
44P Premeiotic arrest	*ZFX*	chrX:24197706T>A *c.465T>A* *g.30417T>A*	p.His155Gln	Polymorphism: amino acid sequence changed protein features (might be) affected splice site changes	Tolerated(0.2)/probably_damaging(0.978)/0.293/ 21.4	rs149552647	European (non‐Finnish): 0.001067 Total: 0.001475	OTHER KNOWN MUTATIONS (H): NM_001178084.1c.2187G>T (p.Arg729Ser) NONOBSTUCTIVE AZOOSPERMIA (meiotic arrest) MGI ID: 2653287 Reproductive system: OLIGOZOOSPERMIA; decreased germ cell number (J:41131)
56P Postmeiotic arrest	*KCND1*	chrX:48823056G>C *c.1396C>G* *g.4921C>G*	p.Leu466Val	Polymorphism: amino acid sequence changed protein features (might be) affected	Tolerated(0.43)/benign(0.007)/2.056/10.51	rs3027482	European (non‐Finnish): 0.01147 Total: 0.006666	MGI ID:5708278 Reproductive system: N/A
P25b N/A	*KCND1*	chrX:48820063A>C *c.1723T>G* *g.7914T>G*	p.Ser575Ala	Polymorphism: amino acid sequence changed protein features (might be) affected splice site changes	Tolerated(0.11)/benign(0.012)/6.22/19.14	rs142098952	European (non‐Finnish): 0.005611 Total: 0.002934	
60P SCOS	*KIAA1210*	chrX:118220986C>A *c.4207G>T* *g.63557G>T*	p.Ala1403Ser	Polymorphism: amino acid sequence changed splice site changes	Tolerated(0.06)/benign(0.282)/−0.412/8.510	rs201193669	European (non‐Finnish): 0.003138 Total: 0.001977	MGI (M): N/A
P108/2015 Postmeiotic arrest	*KIAA1210*	chrX:118230509C>T *c.1214G>A* *g.54034G>A*	p.Arg405His	Polymorphism: amino acid sequence changed splice site changes	Deleterious(0.01)/probably_damaging(0.95)/−0.36/22.2	rs145929840	European (non‐Finnish): 0.01461 Total: 0.009424	
53/P213 MA arrest	*DHRSX*	chrX:2406779T>G *g.14068A>C*	intron	Polymorphism: protein features (might be) affected splice site changes	Deleterious(0.01)/benign(0.198)/3.025/19.6	–	European (non‐Finnish): 0.001366 Total: 0.002170	TRANSLOCATION (H): (DHRSX→ ASMT, SPRY3 →IL9R)×3 male infertility [58]
65P SCOS	*ZMYM3*	chrX:70466490G>C *c.2367C>G* *g.8507C>G*	p.Asn789Lys	Polymorphism: amino acid sequence changed protein features (might be) affected splice site changes	Tolerated(0.63)/benign(0.005)/0.823/14.05	rs151152741	European (non‐Finnish): 0.01188 Total: 0.008666	Zmym3 mice KO: male infertility [59]
P78/2014 N/A	*FAM47C*	***chrX:37028309A>G*** ***c.1826A>G*** ***g.1878A>G***	p.Glu609Gly	Polymorphism: amino acid sequence changed splice site changes	Deleterious(0.04)benign(0.006)/−0.002/12.66	‐	**Variant not found**	OTHER MUTATION (H): chrX:37028866 (rs140378751) Severe oligozoospermia MGI (M): N/A
2L Postmeiotic arrest	*FANCB*	*chrX:14863136A>G* *c.1769T>C* *g.28056T>C*	p.Phe590Ser	Polymorphism: amino acid sequence changed protein features (might be) affected splice site changes	Tolerated(0.05)/benign(0.054)/4.666/17.41	rs142959373	European (non‐Finnish): 0.001191 Total: 0.001462	MGI ID:5688399 Reproductive system: male infertility (J:224588)
9L Postmeiotic arrest	*FAM50B*	*chr6:3850338G>A HOMO* *c.293G>A* *g.719G>A*	p.Arg98Gln	Polymorphism: amino acid sequence changed splice site changes	Tolerated(0.72)/benign(0.007)/0.713/7.369	rs117488732	European (non‐Finnish): 0.01583 (only 18 HOMO) Total: 0.009323 (only 19 HOMO)	MGI ID:6333013 Reproductive system: N/A

*Note*: Ultra‐rare variants (<0.001) are shown in bold. H, human; M, mouse; MGI, mouse genome informatics.

Additionally, we found hemizygous rare variants in individual samples that might be potential NOA‐associated SNVs. In particular, the following variants should be highlighted: c.1723T>G in the *KCND1* gene (*locus* Xp11.23), c.1214G>A in the *KIAA1210* gene (*locus* Xq24), and c.1826A>G in the *FAM47C* gene (*locus* Xp21.1).

All identified rare variants were observed separately in 5 out of 6 patients in whom previously performed WES analysis did not detect any potentially causative variants.

### 3.3 Rare variants in genes not previously associated with male infertility

We also identified a few rare variants in X‐linked genes that have never been investigated with respect to male infertility but could be important in patients with NOA (Table 3). In two patients with SCOS (P13L, P32L), we observed two such variants: c.2753_2754in and c.2617G>C in the *ALG13* gene (*locus* Xq23). Dysfunction of this gene is associated with many clinical symptoms, including endocrine abnormalities,^62^ which can further influence the reproductive tract (Table 3). Moreover, we observed variants in genes that play a role in chromatin modifications and may also be important with respect to spermatogenesis: c.1729A>C and g.93537G>A in the *BRWD3* gene (*locus* Xq21.1)^63^ in patients with NOA (P9L, P65P) and c.1945T>C in the *BEND2* gene (*locus* Xp22.13)^64^ in patients with postmeiotic arrest (Table 3). In another NOA individual (P63/P219), we identified variant c.544T>C in the *FAM47B* gene (*locus* Xp21.1), the function of which is unknown, but another member of this gene family, *FAM47C*, is associated with severe oligozoospermia. ^59^ In addition, c.301C>T in the *FAM9B* gene and c.391G>A in the *FAM9C* gene (*locus* Xp22.2) were identified (Table 3). The exact role of the *FAM9B* and *FAM9C* genes is unknown; however, studies have indicated that they may be involved in the meiotic process.^65^ Additionally, *FAM9B* is associated with serum testosterone concentration^66^ (Table 3). We also focused attention on genes from the *MAGE* family, which have been described in the development of malignances, that is, the *MAGEB6* gene (*locus* Xp21.3) but have never been reported in azoospermia. Expression of the *MAGEB6* gene is normally specific only for gametogenic tissues;^67^ therefore, it is worth studying it from spermatogenetic aspects. We identified the following *MAGEB6* variants: c.293G>A, c.299C>T, and c.1162C>T in three patients with NOA (P32L, P54L, P57P) (Table 3). The *MAP3K15* (*locus* Xp22.12) gene, which is expressed at low levels in the testis, may indirectly affect spermatogenesis because it is involved in testicular steroidogenesis.^68^ We found different variants in this gene in three patients with NOA: P34P and P254 variant c.3751C>G, while in the case of P45L variant, we observed c.596G>A (Table 3).

**TABLE 3:** Identified rare variants in genes not previously linked with male infertility

						**Controls from dbSNP database**		
**Patient no/phenotype**	**Gene**	**Change in DNA (according to GRCh37)**	**Protein/RNA change**	**Mutation taster prediction**	**SIFT/PolyPhen/ PhyloP100/ CADD**	SNP	Allele frequency GnomAD	**Chromosomal translocations (H)/other known mutations (H)/pYmouse KO‐ MGI (M)**
Variants in genes not related with infertility/spermatogenesis								
13L SCOS	*ALG13*	**chrX:110987953_110987954insACC** ***c.2753_2754insACC*** ***g.78911_78912insACC***	insertion of 1 AA p.Pro944_Pro945dup	Polymorphism: amino acid sequence changed protein features (might be) affected splice site changes	–/–/–/–	–	**European (non‐Finnish): 0.0002189** **Total: 0.0003000**	MGI ID:6358168 Reproductive system: N/A
32L SCOS	*ALG13*	chrX:110980029G>C *c.2617G>C* *g.70987G>C*	p.Ala873Pro	Polymorphism: amino acid sequence changed protein features (might be) affected splice site changes	Tolerated(0.06)/ benign(0.006)/ 0.219/8.052	rs142841538	European (non‐Finnish): 0.002132 Total: 0.002167	
64L Postmeiotic arrest	*BEND2*	**chrX:18192186A>G** ***c.1945T>C*** ***g.46839T>C***	p.Ser649Pro	Polymorphism	Deleterious(0.03)/possibly_damaging(0.879)/–0.14/13.24	rs145726572	**European (non‐Finnish): 0.0008982** **Total: 0.0007136**	MGI: N/A
9L Postmeiotic arrest	*BRWD3*	chrX:79978208T>G *c.1729A>C* *g.86980A>C*	p.Thr577Pro	Disease causing: amino acid sequence changed	Deleterious(0)/probably_damaging(0.997)/7.482/25.4	–	**Variant not found**	MGI ID:3867498 Reproductive system: N/A
65P SCOS	*BRWD3*	chrX:79971651C>T *g.93537G>A*	intron	Polymorphism	–/–/6.083/22.2	rs186391561	European (non‐Finnish): 0.008962 Total: 0.006290	
41P Postmeiotic arrest	*DDX53*	chrX:23018493G>T *c.319G>T* *g.407G>T*	p.Ala107Ser	Polymorphism: amino acid sequence changed protein features (might be) affected	Tolerated (0.55)/benign (0.079)/0.334/0.099	rs148588561	European (non‐Finnish): 0.004073 Total: 0.001995	N/A
33P Postmeiotic arrest	*TAF4*	**chr20:60640266T>G** **HOMO** ***c.601A>C*** ***g.601A>C***	p.Ser201Arg	Disease causing: amino acid sequence changed	Tolerated (0.09)/ benign (0.079)/2.112/17.16	–	**Variant not found**	MGI ID:2152346 Reproductive system: N/A
63/P219 Postmeiotic arrest	*FAM47B*	chrX:34961492T>C *c.544T>C* *g.580T>C*	p.Tyr182His	Polymorphism: amino acid sequence changed	Tolerated (0.26)/benign(0.01)/0.04 /2.725	rs147688579	European (non‐Finnish): 0.01135 Total: 0.008269	MGI: N/A
48P Postmeiotic arrest	*FAM9B*	chrX:8997440G>A *c.301C>T* *g.135241C>T*	p.His101Tyr	Polymorphism: amino acid sequence changed	Tolerated(1)/possibly_damaging(0.728)/‐0.21/2.927	rs141078293	European (non‐Finnish): 0.01125 Total: 0.006876	MGI: N/A
32L SCOS	*FAM9C*	**chrX:13057974C>T** ***c.391G>A*** ***g.4828G>A***	p.Asp131Asn	Polymorphism: amino acid sequence changed splice site changes	Deleterious (0.03)/benign (0.015)/1.399/12.76	–	**Variant not found**	MGI: N/A
32L SCOS	*MAGEB6*	**chrX:26212256G>A** ***c.293G>A*** ***g.1700G>A***	p.Arg98His	Polymorphism: amino acid sequence changed protein features (might be) affected	Tolerated (0.09)/benign (0)/−3.633/0.001	rs4630029	**European (non‐Finnish): 0.00005073** **Total: 0.00005850**	MGI: N/A
		**chrX:26212262C>T** ***c.299C>T*** ***g.1706C>T***	p.Ala100Val	Polymorphism: amino acid sequence changed protein features (might be) affected splice site changes	Deleterious (0.02)/benign(0.385)/−0.037/ 13.50	rs4272533	**European (non‐Finnish): 0.000** **Total: 0.00001705**	
54L N/A	*MAGEB6*	**chrX:26212256G>A** ***c.293G>A*** ***g.1700G>A***	p.Arg98His	Polymorphism: amino acid sequence changed protein features (might be) affected	Tolerated (0.09)/benign (0)/−3.633/ 0.001	rs4630029	**European (non‐Finnish): 0.00005073** **Total: 0.00005850**	
57P Postmeiotic arrest	*MAGEB6*	chrX:26213125C>T *c.1162C>T* *g.2569C>T*	p.Pro388Ser	Polymorphism: amino acid sequence changed protein features (might be) affected splice site changes	Deleterious (0.01)/probably_damaging(0.979)/−0.948/15.74	rs147228278	European (non‐Finnish): 0.01049 Total: 0.007744	
Variants in genes not expressed in testis								
34P Premeiotic arrest	*MAP3K15*	chrX:19379640G>C *c.3751C>G* *g.153740C>G*	p.Gln1251Glu	Polymorphism: amino acid sequence changed splice site changes	Tolerated (0.27)/benign (0.433)/5.57/16.47	rs15943	European (non‐Finnish): 0.006453 Total: 0.003767	MGI ID: 5644160 Reproductive system: N/A
45L Postmeiotic arrest	*MAP3K15*	chrX:19482454C>T *c.596G>A* *g.50926G>A*	p.Ser199Asn	Polymorphism: amino acid sequence changed protein features (might be) affected splice site changes	Tolerated (0.26)/benign (0.025)/1.34/10.09	rs55916006	European (non‐Finnish): 0.006326 Total: 0.005334	
P254 N/A	*MAP3K15*	chrX:19379640G>C *c.3751C>G* *g.153740C>G*	p.Gln1251Glu	Polymorphism: amino acid sequence changed splice site changes	Tolerated (0.27)/benign (0.433)/5.57/16.47	rs15943/ not specified	European (non‐Finnish): 0.006453 Total: 0.003767	
Variants in genes with no known function								
50L SCOS	*RBMXL3*	chrX:114426292G>A *c.2288G>A* *g.2330G>A*	p.Gly763Asp	Polymorphism: amino acid sequence changed protein features (might be) affected	Tolerated (0.13)/probably_damaging(0.992)/2.158/14.95	rs199838194/ Not Reported in ClinVar	European (non‐Finnish): 0.001610 Total: 0.001150	N/A
64L SCOS	*RBMXL3*	chrX:114424734C>T *c.730C>T* *g.772C>T*	p.Pro244Ser	Polymorphism: amino acid sequence changed protein features (might be) affected splice site changes	Tolerated (0.06)/benign (0.313)/0.109/1.791	rs184389455/ Not Reported in ClinVar	European (non‐Finnish): 0.01743 Total: 0.009564	
13L SCOS	*SSX3*	chrX:48209429C>T *c.459G>A* *g.6714G>A*	p.Met153Ile	Polymorphism: amino acid sequence changed splice site changes	Tolerated (0.08)/benign(0)/−1.684/8.519	rs148463135/not reported in ClinVar	European (non‐Finnish): 0.003219 Total: 0.002215	N/A
65P SCOS	*SSX3*	chrX:48207027C>G *g.9116G>C*	intron	amino acid s Polymorphism: equence changed splice site changes	Tolerated (0.14)/benign (0.003)/−2.175/8.519	rs145941341/Not Reported in ClinVar	European (non‐Finnish): 0.006055 Total: 0.004350	
P108/2015 N/A	*FMR1NB*	**chrX:147088321C>T** ***c.497C>T*** ***g.25473C>T***	p.Ser166Leu	Polymorphism: amino acid sequence changed protein features (might be) affected splice site changes	Tolerated (0.14)/benign (0.003)/−1.077/0.044	‐	**European (non‐Finnish): 0.0001191** **Total: 0.00006378**	MGI ID:6194712 Reproductive system: normal (J:234235)

*Note*: Ultra‐rare variants (<0.001) are shown in bold. H, human; M, mouse; MGI, mouse genome informatics.

Finally, we would like to draw attention to the genes that have never been studied with respect to azoospermia and whose function has not yet been known but they were identified using WGS. These genes are X‐linked and are exclusively expressed in the testis; therefore, the identified rare variants could be associated with azoospermia. We observed rare variants: c.2288G>A and c.730C>T in the *RBMXL3* gene (*locus* Xq23) in two patients with SCOS (P50L, P64L) (Table 3). We also identified two additional rare variants: c.459G>A, g.9116G>C in the *SSX3* gene (*locus* Xp11.23) in patients with SCOS (P13L, P65P); and variant chrX:147088321C>T in the *FMR1NB* gene (*locus* Xq27.3‐q28) in patients with postmeiotic arrest (Table 3).

## 4 DISCUSSION

The NOA phenotype is a complex type of infertility, and still little is known about the genetic causes that may lead to spermatogenetic failure. Here, we present candidates of NOA‐associated rare SNVs determined by WGS that in various combinations might be implicated in the molecular background of male infertility. Some of the genes with identified variants have never been studied in NOA. However, it is worth paying them special attention since their expression is mostly in the testis and/or endocrine system and their potential function may directly/or indirectly be associated with spermatogenesis.

One notable WGS finding is variants in a novel candidate gene *TKTL1*, in which were identified likely pathogenic variants c.268_268delG and c.1601A>G (Table 1, Figure S2). Homology models were used to predict the functional implications of mutations of interest. First variant c.268_268delG–p.Asp90Met fs*35 not only results in the mutation of a negatively charged aspartic acid residue to a hydrophobic amino acid but also truncates most of the protein, presumably eliminating protein sequence and its activity (Figure 1A). In addition, while variant c.1601A>G–p.Glu534Gly resides within the C‐terminal domain that does not comprise the active site, and the glutamic acid at position 534 is predicted to stabilize the fold of the domain via two hydrogen bonds with the backbone residues of p.IleI542 and p.Gly543 (Figure 1A). In addition, homodimerization is in part mediated by the C‐terminal domain, suggesting that a mutation that destabilizes the fold of this domain could destabilize the quaternary structure of the functional protein (Figure 1). TKTL1 is an enzyme involved in the nonoxidative pentose‐phosphate pathway that was reported to be overexpressed in several human cancers.^69^ In the proteomic analysis, TKTL1 was demonstrated to be a biomarker that could distinguish between semen from fertile and NOA men.^70^ Based on this information and the similarities between gametogenesis and carcinogenesis, we suggest that the *TKTL1* gene may also be important in spermatogenetic processes.

Moreover, we identified novel rare variants (c.1184T>G, c.758A>G, g.114567C>G, c.2554T>G) within the *IGSF1* gene in 4 out of 39 patients with NOA (Table 1). Homology models allowed us to preliminarily interpret the physical consequences of changing the amino acids of particular patient variants. For example, the position of L395 in the homology model suggests that mutation to an arginine would not be accommodated in the space supporting the protein fold (Figure 1A); therefore, we predict that mutation p.Leu395Arg would deform the fold and likely the surface of this domain, possibly affecting its interactions with surrounding domains and with other molecules. p.Tyr253 and p.Tyr838 have been modeled on the surface of two Ig folds with their side chains oriented toward the solution. This suggests that mutations p.Tyr253Cys and p.Tyr838Asp affect interactions with other domains and/or molecules, including ligands of the receptor (Figure 1A). In three of these variants, in silico analysis revealed their possible disease‐causing effect. *Igsf1* deletion decreases the production of pituitary hormones and circulating TSH in mice, perhaps secondary to impaired thyrotropin‐releasing hormone (TRH) receptor signaling.^42^ Loss of the IGSF1 function is associated with profound hypothyroxinemia in some patients.^42^ Other studies have shown a possible association between an *IGSF1* mutation and neurological phenotypes; however, other phenotypic consequences were also observed within the affected family, including macroorchidism and infertility.^71^ This indicates that the rare variants identified in this study within the *IGSF1* gene may also be closely related to azoospermia phenotype.

The next interesting novel disease‐causing candidate variants found in this study is the *ZFPM2* gene (Table 1). Intrinsically disordered regions (IDRs) of proteins are not simply unstructured; they have charge and hydrophobicity patterns that can facilitate intermolecular interactions, structure formation upon binding, and functional phase separation.^72^, ^73^ Since both p.Glu30 and p.Val763 are predicted to be embedded in long, low‐complexity disordered regions (Figure S3), assessing the effects of p.Glu30Gly and p.Val763Ile is difficult. Regarding p.Glu30Gly, it is possible that p.Glu30 interacts with basic histone proteins, and mutation to glycine does not allow for the same recognition of chromatin. IDRs of proteins also have characteristic charge pattern, which alters E30G, potentially affecting its biological and biophysical behavior.^73^ Interestingly, it has been reported that this natural variant does not affect the interaction of this protein with GATA4 in patients with heart conditions,^74^, ^75^ suggesting a distinct molecular network for this protein in fertility. That phenotype is observed with the conservative change from valine to isoleucine at position 763‐both beta‐branch hydrophobic amino acids suggesting that this residue plays an important role in intermolecular interactions, which might be responsible for its subsequent structure, function, and/or phase‐separation characteristics. Since the position of this amino acid resides between two zinc fingers (Figure S3A,B), its identity may play an important role in the correct orientation of the DNA‐binding elements relative to each other. Some mutations in the *ZFMP2* gene were previously described in a DSD,^46^ but have never been identified in NOA. However, this gene could also be important in spermatogenesis because the DSD leads to infertility.

Another gene with identified variant is *VCX3A*. This variant c.556G>A introduced the amino acid change p.Val186Met (Table 1, Figure 1D). This is a conservative mutation from one to another hydrophobic amino acid and would not be expected to have significant phenotypic implications unless its identity is important for intermolecular interactions or for facilitating the function of adjacent low‐complexity repeats. Members of this protein family have been associated with mRNA stabilization^76^ and ribosomal assembly during spermatogenesis;^77^ therefore, it is possible that this protein becomes ordered upon interaction with mRNA, ribosomal components, or chaperones and that a valine at position 186 is necessary for these associations. Moreover, low expression of the *VCX3A* gene was reported in infertile cryptorchid males,^52^ and deletion of a region including this gene was noted in cases with congenital hypogonadotropic hypogonadism,^78^ which indicates that the identified variant may also be associated with spermatogenesis disruption, leading to azoospermia.

The next group of genes with identified single rare variants was found in sporadic samples with a potential causative effect on spermatogenesis (Table 2). In particular, we noted ultrarare variants of the *ESX1* gene (c.1040C>G, c.1042C>G) and *TEX13A* (c.596G>A) that were detected in patients with postmeiotic arrest (Table 2). Importantly, within the group of NOA patients examined using the WGS method were samples that had been previously tested using WES with no apparent results. In the case of *ESX1* gene, a group from China reported for the first time a rare *ESX1* gene variant unique to Chinese patients with NOA and no mature sperm could be found in the testicular biopsy of those patients.^53^ They also found similar variants (c.1094C>G and c.1096C>G) to ours, which are located in the proline‐rich repeat region, but more closer to the end of this region.^53^ The compound variants compromised the stabilizing effect of ESX1 on cyclin A, thereby causing the failure of M phase arrest in cells.^53^ Therefore, we would predict that also our identified variants, which were analogical to the reported variants would destabilize cyclins, thereby affecting the cell cycle.

The *TKTL1* and *ESX1* genes are currently analyzed by our group using RNA‐seq to create a gene network that can be influenced by these genes to partly determine the biological function in the CRISPR‐modified cells obtained from human male gonad (unpublished, manuscript in preparation).

We also identified several rare variants in genes that have been investigated in the aspect of disease but never in connection with male infertility. Rare variants of *ALG13* genes were detected in two patients with SCOS (Table 3), and it was previously reported that *ALG13* was associated with clinical symptoms, including microcephaly, seizures, hypotonia, mild‐to‐moderate intellectual disability, dystonia, hepatomegaly, coagulopathy, infections, endocrine abnormalities and abnormal secretory protein glycosylation.^62^

We also focused our attention on the *MAGEB6* gene, which was previously described in association with malignancy^79^ but has never been studied in azoospermia. We identified three variants of *MAGEB6* in NOA patients with different degrees of spermatogenetic impairment (Table 3). Such genes code for cancer‐testis associated antigens (CTAs), which are expressed in diverse histological types of malignant tumors but also in immunoprivileged (gametogenic) tissues. Due to some similarities between gametogenesis and tumorigenesis, we suggest that the *MAGEB6* gene may also be important in spermatogenetic processes and connected with cell proliferation and growth.^67^, ^79^

Other genes that attracted our attention exhibited no or low expression in the testis. However, they might affect the spermatogenetic process indirectly, while their identified variants may have a negative impact on this process. One of them was the *MAP3K15* gene, for which we observed variants in three patients, especially in those with postmeiotic arrest (Table 3). The *MAP3K15* gene was previously described to be associated with cell stress signaling; however, recently, it has been reported to be a novel kinase linked to steroidogenesis.^68^ Therefore, we suggest further studies with respect to azoospermia syndrome, in which low levels of steroid hormones may disrupt spermatogenesis.

Most importantly, we would like to highlight genes that have never been studied in azoospermic aspects and whose function is not yet known, such as the *RBMXL3*, *SSX3*, and *FMR1NB* genes (Table 3). These genes are exclusively expressed in the testis, and the variants identified in this study were either noted as low frequency or were not noted in the GnomAD database. Thus, we strongly advise further functional studies of these genes in assessing the likelihood of their effect on male fertility. The problem is that neither *RBMXL3* nor *SSX3* genes have discovered orthologues in rodents, and functional studies can be difficult because it seems that there is no adequate animal model for this purpose, so far.

## 5 CONCLUSIONS

In summary, we revealed novel potential candidate NOA‐associated genes in 29 individuals out of 39 azoospermic males (Table S2). We noted that among identified variants only two (in one case 3) of the 29 individuals shared the same variant—overlaps (Table S2). The 16 out of the 58 rare variants were totally newly discovered SNVs, we noted also novel SNVs in 9 NOA‐associated genes and 10 compound SNVs (Table S2). It is highly recommended to examine their possible function and mechanism of participation in gametogenesis. Such studies with selected rare gene variants should be a subject of future research using the CRISPR technique in germ cell suspensions of testicular origin to determine their significance in spermatogenesis and male reproductive health. Furthermore, from a long‐term perspective, delineating genes critical for spermatogenesis may pave the way for genetic correction using a gene editing approach.

## AUTHOR CONTRIBUTIONS

Agnieszka Malcher designed the study and drafted the manuscript, performed gene selection for WGS, oversaw task coordination and supervision, data collection and interpretation, and funding. Tomasz Stokowy and Dawid Sielski performed bioinformatics analysis of WGS. Andrea Berman performed protein modeling and interpretation. Marta Olszewska prepared samples and performed data interpretation. Piotr Jedrzejczak recruited patients and reviewed their medical history. Adam Nowakowski recruited patients. Natalia Rozwadowska collected data. Alexander N. Yatsenko interpreted WES results and helped with editing and finalizing the manuscript. Maciej Kurpisz recruited patients, collected their medical history, and helped with editing and finalizing the manuscript.

All authors have read the manuscript and accepted its final version.

## CONFLICT OF INTEREST

The authors declare that there is no conflict of interest that could be perceived as prejudicing the impartiality of the research reported.

## Supporting information


**Figure S1** Scheme of variant filtering in whole genome‐sequencing analysis

**Figure S2** Experimental verification for *TKTL1* gene with: (A) histopathological images for the patients with identified single nucleotide variants (SNVs); (B) results of bam file from Whole genome sequencing (WGS) and Sanger sequencing to present the SNVs; (C) gene expression level of *TKTL1* using qPCR (***p<0.001); (D) protein expression pattern of TKTL1 using Western blot

**Figure S3** (A) Linear organization of VCX3A, ZFPM2, and IGSF1. ZnF, zinc finger; PR‐SET, PR‐SET domain. The eight L–S–Q–E–S–[E or Q]–V–E–E–P sequence motifs of VCX3A are shown as light gray boxes. Below, residues of interest are indicated along the linear diagram. (B) Probability of disorder for each residue shown for VCX3A (gray), ZFPM2 (orange), and IGSF1 (blue) as calculated by IUPred2A^49,50^. Residues of interest are indicated. Note that the diagrams in A and B are to scale, such that the folded domains can be observed as having a low probability of disorder in panel (B)

**Table S1** Histopathological description of testicular biopsy samples and hormonal levels in patients recruited to the study

**Table S2** Summary of detected variants in NOA patients

**Table S3** Single nucleotide variant (SNV) in known genes which mutation lead to spermatogenetic failure

**Table S4** Information about studied genes

## Data Availability

All data generated or analyzed during this study are included in this published article and its supplemental information files. The results described in the publication are based on whole‐genome sequencing data which includes sensitive information in the form of patient specific germline variants. Information regarding such variants must not be shared publicly following the European Union legislation described in the following document: https://publications.jrc.ec.europa.eu/repository/bitstream/JRC113479/policy_report_‐_review_of_eu_national_legislation_on_genomics_‐_with_identifiers_1.pdf, therefore the access to raw data that support findings of this study are available from the corresponding author upon reasonable request.
