# Supplementary figures and images for: Whole‐genome sequencing identifies new candidate genes for nonobstructive azoospermia
Source: Andrology. 2022 Sep 7;10(8):1605–24. doi: 10.1111/andr.13269 (PMC9826517; doi:10.1111/andr.13269)

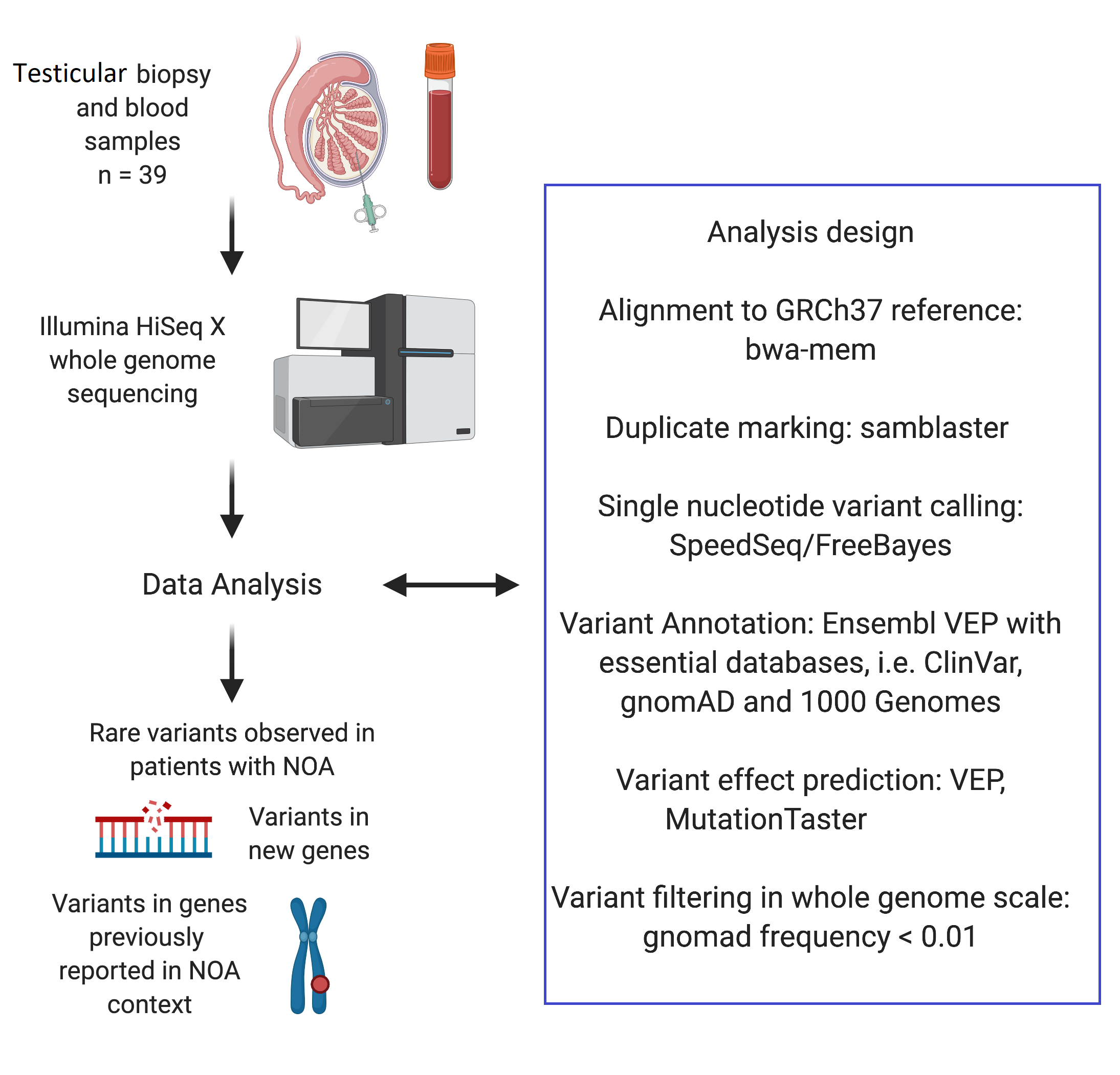

Supplement: Supplementary file 1 — Figure S1 Scheme of variant filtering in whole genome‐sequencing analysis [file ANDR-10-1605-s004.tif]

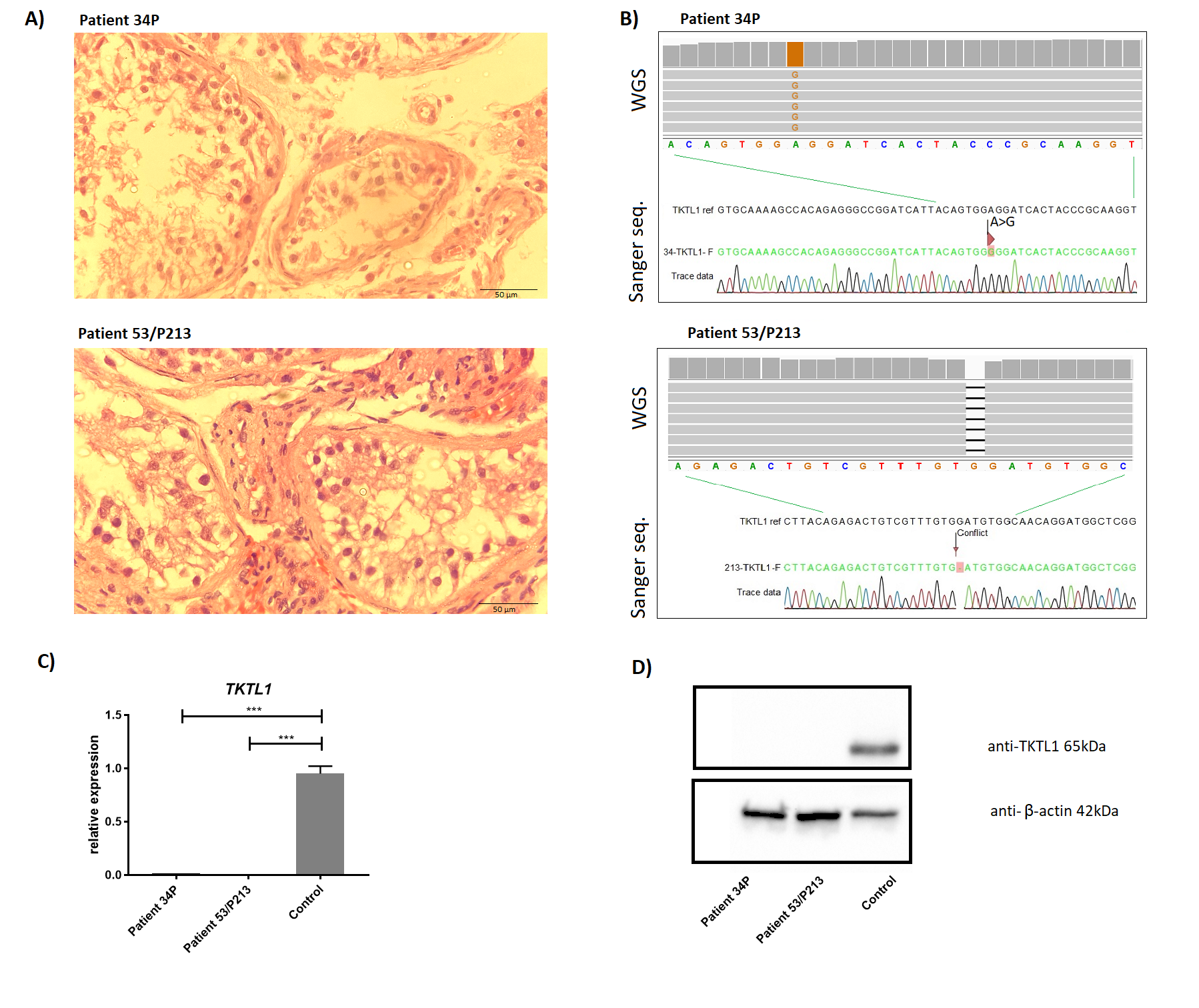

Supplement: Supplementary file 2 — Figure S2 Experimental verification for TKTL1 gene with: (A) histopathological images for the patients with identified single nucleotide variants (SNVs); (B) results of bam file from Whole genome sequencing (WGS) and Sanger sequencing to present the SNVs; (C) gene expression level of TKTL1 using qPCR (***p<0.001); (D) protein expression pattern of TKTL1 using Western blot [file ANDR-10-1605-s001.tif]

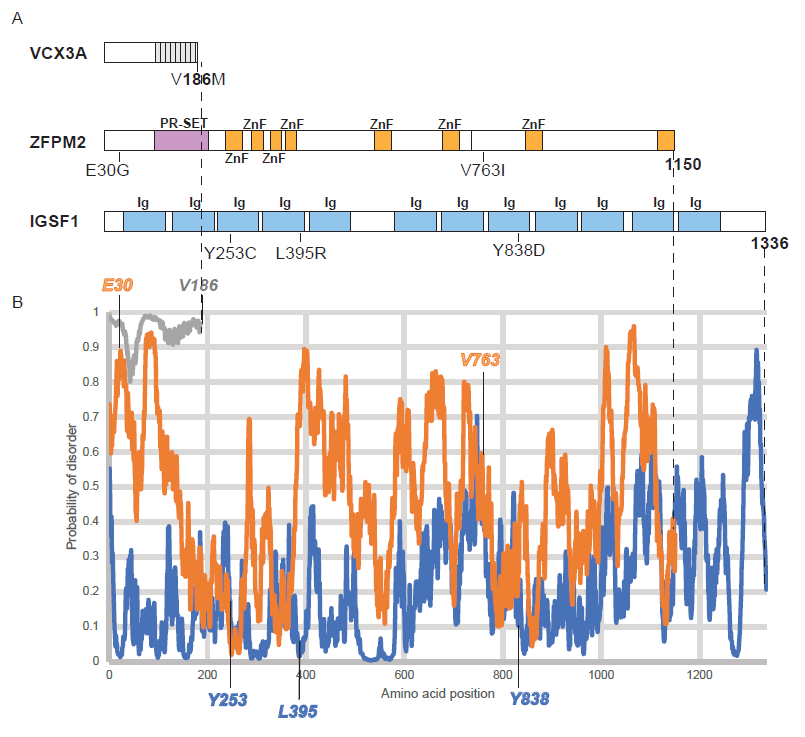

Supplement: Supplementary file 3 — Figure S3 (A) Linear organization of VCX3A, ZFPM2, and IGSF1. ZnF, zinc finger; PR‐SET, PR‐SET domain. The eight L–S–Q–E–S–[E or Q]–V–E–E–P sequence motifs of VCX3A are shown as light gray boxes. Below, residues of interest are indicated along the linear diagram. (B) Probability of disorder for each residue shown for VCX3A (gray), ZFPM2 (orange), and IGSF1 (blue) as calculated by IUPred2A49,50. Residues of interest are indicated. Note that the diagrams in A and B are to scale, such that the folded domains can be observed as having a low probability of disorder in panel (B) [file ANDR-10-1605-s006.tif]
