# Supplementary material for: Whole‐genome sequencing identifies new candidate genes for nonobstructive azoospermia
Source: Andrology. 2022 Sep 7;10(8):1605–24. doi: 10.1111/andr.13269 (PMC9826517; doi:10.1111/andr.13269)
Supplement: Supplementary file 4 — Table S1 Histopathological description of testicular biopsy samples and hormonal levels in patients recruited to the study [file ANDR-10-1605-s002.docx]

**Supplemental Table 1.** Histopathological description of testicular biopsy samples and hormonal levels in patients recruited to the study.

|  | ID sample | Age | Semen analysis/Histopathological description | Johnsen score | Hormonal level |
| --- | --- | --- | --- | --- | --- |
| 1 | 2L | 28 | Azoospermia  Postmeiotic arrest: Slightly impaired spermatogenesis, numerous late spermatids, disorganized epithelium | 9 | LH:2.59 ng/ml  FSH:3.21 ng/ml  E_2_:21.70 ng/ml  PRL:4.05 ng/ml  T:352 ng/dl |
| 2 | 9L | 30 | Azoospermia  Postmeiotic arrest: Less than five spermatozoa per tubule, few late spermatids | 8 | FSH:34.4 ng/ml  T:8.11 mmol |
| 3 | 10L | 45 | Azoospermia  Postmeiotic arrest: Less than five spermatozoa per tubule, few late spermatids | 8 | N/A |
| 4 | 13L | 31 | Azoospermia  SCOS: No germ cells, Sertoli cells only | 2 | FSH:7.45 ng/ml  T:402 ng/dl |
| 5 | 31L | 29 | Azoospermia  SCOS: No germ cells, Sertoli cells only | 2 | FSH:10.5 ng/ml  LH:6.0 ng/ml  PRL:255 ng/dl  T:5.7 mmol  E_2_: 34.2 ng/ml |
| 6 | 32L | 31 | Azoospermia  SCOS: No germ cells, Sertoli cells only | 2 | FSH:7.89 ng/ml  PRL:15.29 ng/ml  T:4.46 mmol  TSH:6.3 ng/ml  LH:3.69 ng/ml |
| 7 | 33P | 34 | Azoospermia  Postmeiotic arrest: Less than five spermatozoa per tubule, few late spermatids | 8 | FSH:19.64mIU/ml H  LH:6.38 mIU/ml  E_2_:17.0 mIU/ml  PRL:11.43mIU/ml  T:430.0 ng/dl |
| 8 | 34P | 35 | Azoospermia  Premeiotic arrest: Spermatogonia only | 3 | FSH:10.34mIU/ml N  T:417ng/dl  LH:5.5mIU/ml |
| 9 | 37L | 39 | Azoospermia  Postmeiotic arrest: Slightly impaired spermatogenesis, numerous late spermatids, disorganized epithelium | 9 | FSH:27mIU/ml  T:5.2ng/ml |
| 10 | 38L | 33 | Azoospermia  MA arrest: No spermatozoa or spermatids, few spermatocytes | 4 | N/A |
| 11 | 41P | 23 | Azoopermia  MA arrest: No spermatozoa or spermatids, many spermatocytes | 5 | N/A |
| 12 | 42P | 37 | Azoospermia  Postmeiotic arrest | 9 | FSH:2.6 ng/ml  T:5.25 mmol |
| 13 | 43P | 28 | Azoospermia  SCOS: No germ cells, Sertoli cells only | 2 | FSH:14.7 ng/ml  LH:8.1 ng/ml  PRL:16.8ng/ml  T:6 mmol |
| 14 | 44P | 25 | Azoospermia  Premeiotic arrest: Spermatogonia only | 3 | FSH:1.9 mIU/ml  T:4.9 mmol |
| 15 | 45L | 29 | Azoospermia  Postmeiotic arrest: Slightly impaired spermatogenesis, numerous late spermatids, disorganized epithelium | 9 | FSH:20.9 ng/ml  LH:4.3 ng/ml N  PRL:12.03 ng/ml  T:16.6 mmol N |
| 16 | 46L | 36 | Azoospermia  Postmeiotic arrest: Slightly impaired spermatogenesis, numerous late spermatids, disorganized epithelium | 9 | FSH:0.35mIU/ml  LH:0.13 mIU/ml  T:19.22 mmol |
| 17 | 48P | 29 | Azoospermia  Postmeiotic arrest: Slightly impaired spermatogenesis, numerous late spermatids, disorganized epithelium | 9 | FSH:2.45mIU/ml  LH:5.35mIU/ml  T:28mmol/l |
| 18 | 49P | 32 | Azoospermia  Premeiotic arrest: Spermatogonia only | 3 | TSH:2.3 mIU/ml  FSH:10.7 mIU/ml  LH:5.4 mIU/ml  PRL:396 mIU/ml  T:9.29mmol |
| 19 | 50L | 42 | Azoospermia  SCOS: No germ cells, Sertoli cells only | 2 | N/A |
| 20 | 52 | N/A | Azoospermia  SCOS: No germ cells, Sertoli cells only | 2 | N/A |
| 21 | 53/P213 | 30 | Azoospermia  MA arrest: No spermatozoa or spermatids, few spermatocytes | 4 | FSH:23 mIU/ml  LH:8.8 mIU/ml  T:280ng/dl |
| 22 | 54L | 30 | Azoospermia | N/A | N/A |
| 23 | 56P | 30 | Azoospermia  No spermatozoa, no late spermatids, few early spermatids | 7 | T:4.8ng/ml  FSH:4.7 ng/ml |
| 24 | 57P | 31 | Azoospermia  Postmeiotic arrest: Slightly impaired spermatogenesis, many late spermatids, disorganized epithelium | 9 | T:600ng/dl  FSH:2 ng/ml |
| 25 | 58P | 40 | Azoospermia  Postmeiotic arrest: Less than five spermatozoa per tubule, few late spermatids | 8 | FSH:17.3 mIU/ml  LH:5.6 mIU/ml  PRL: 17.5 mIU/ml  T:582.9 ng/dl |
| 26 | 59P | 30 | Azoospermia  Postmeiotic arrest: Less than five spermatozoa per tubule, few late spermatids | 8 | FSH:5.8 mIU/ml  T:370ng/dl |
| 27 | 60P | 29 | Azoospermia  SCOS: No germ cells, Sertoli cells only | 2 | FSH:14mIU/ml  LH:11mIU/ml  T:129ng/dl |
| 28 | 61L | 31 | Azoospermia  SCOS: No germ cells, Sertoli cells only | 2 | FSH:25.1mIU/ml  LH:6.4 mIU/ml  T:529.5ng/dl  PRL:1.9mIU/ml |
| 29 | 63P/P219 | 41 | Azoospermia  Postmeiotic arrest: No spermatozoa, no late spermatids, few early spermatids | 6 | FSH:2.45mIU/ml LH:5.35mIU/ml T:28mmol/l |
| 30 | 64L | 32 | Azoospermia  SCOS: No germ cells, Sertoli cells only | 2 | FSH:3.7mIU/ml  T:433ng/dl |
| 31 | 65P | 31 | Azoospermia  SCOS: No germ cells, Sertoli cells only | 2 | FSH:28mIU/ml  LH:10mIU/ml  T:571mIU/ml |
| 32 | 66L | 39 | Azoospermia  Postmeiotic arrest: Less than five spermatozoa per tubule, few late spermatids | 8 | FSH:3.6mIU/ml  LH:2.9mIU/ml  T:561ng/dl |
| 33 | P25b | N/A | Azoospermia | N/A | N/A |
| 34 | P34/2012 | N/A | Azoospermia | N/A | N/A |
| 35 | P76/2014 | N/A | Azoospermia | N/A | N/A |
| 36 | P78/2014 | N/A | Azoospermia | N/A | N/A |
| 37 | P108/2015 | N/A | Azoospermia | N/A | N/A |
| 38 | P235 | 33 | Azoospermia  Postmeiotic arrest: Less than five spermatozoa per tubule, few late spermatids | 8 | FSH:20.5mIU/ml  LH:3,9mIU/ml T:2.28 ng/ml  PRL:21,54 mIU/ml |
| 39 | P254 | 40 | Azoospermia | N/A | FSH: 9.9mIU/ml  LH 8.7 mIU/ml  T- 387 ng/dl  PRL- 8.1 mIU/ml |

*Samples of which mutations in WES were not found marked in blue color*
